# Berberine Improves Glucose and Lipid Metabolism in HepG2 Cells Through AMPKα1 Activation

**DOI:** 10.3389/fphar.2020.00647

**Published:** 2020-05-08

**Authors:** Gang Ren, Jiang-Hong Guo, Yu-Zhen Qian, Wei-Jia Kong, Jian-Dong Jiang

**Affiliations:** ^1^Department of Virology, Institute of Medicinal Biotechnology, Chinese Academy of Medical Sciences and Peking Union Medical College, Beijing, China; ^2^State Key Laboratory of Bioactive Substance and Function of Natural Medicines, Institute of Materia Medica, Chinese Academy of Medical Sciences and Peking Union Medical College, Beijing, China; ^3^School of Life Sciences, Liaoning Normal University, Dalian, China

**Keywords:** berberine, AMP-activated protein kinase α1, glucose consumption, gluconeogenesis, lipogenesis

## Abstract

**Aim:**

This study is designed to investigate whether or not AMP-activated protein kinase α1 (AMPKα1) is required for natural product berberine (BBR) to improve glucose and lipid metabolism in HepG2 cells.

**Methods:**

AMPKα1 knocked-out (KO, *AMPKα1^-/-^*) cells were obtained by co-transfection of the CRISPR/Cas9 KO and HDR (homology-directed repair) plasmid into HepG2 cells, as well as subsequent screen with puromycin. The expression levels of target proteins or mRNAs were determined by western blot or real-time RT-PCR, respectively. Cellular AMPK activity, glucose consumption, lactate release, glucose production, and lipid accumulation were determined by kits.

**Results:**

The results showed that the *AMPKα1* gene was successfully KO in HepG2 cells. In *AMPKα1^-/-^* cells, the protein expression of AMPKα1 and phosphorylated-AMPKα1 (p-AMPKα1) disappeared, the level of total AMPKα declined to about 45–50% of wild type (*p* < 0.01), while p-AMPKα level and AMPK activity were reduced to less than 10% of wild type (*p* < 0.001). BBR increased p-AMPKα1, p-AMPKα, AMPK activity, and stimulated glucose consumption, lactate release, inhibited glucose production in wild type HepG2 cells (*p* < 0.05 or *p* < 0.01). BBR also reduced intracellular lipid accumulation and suppressed the expression of lipogenic genes in oleic acid (OA) treated wild type HepG2 cells (*p* < 0.05 or *p* < 0.01). In *AMPKα1^-/-^* HepG2 cells, the stimulating effects of BBR on p-AMPKα1, p-AMPKα, AMPK activity, and its improving effects on glucose and lipid metabolism were completely abolished.

**Conclusion:**

Our study proves that AMPKα1 plays a critical role for BBR to improve glucose and lipid metabolism in HepG2 cells. Our results will provide new information to further understand the molecular mechanisms of BBR.

## Introduction

The cellular AMP-activated protein kinase (AMPK) plays a central role in the modulation of energy balance of metabolic tissues by suppressing anabolism and promoting catabolism (Steinberg and Kemp, 2009). Now AMPK is considered as an important molecular target for the treatment of metabolic diseases such as diabetes, obesity, or dyslipidemia (Steinberg and Kemp, 2009).

Berberine (BBR) is a natural compound isolated from herbal medicines such as *Coptis chinensis* and goldenseal with multiple pharmacological activities. It has been identified to be an effective hypoglycemic and hypolipidemic agent in clinic (Kumar et al., 2015; Yao et al., 2015). BBR improves glucose and lipid metabolism through multiple targets, which include the AMPK (Kim et al., 2009; Kumar et al., 2015; Yao et al., 2015). BBR was demonstrated to activate AMPK in liver cells, skeletal muscle cells, and adipocytes, perhaps through inhibiting mitochondrial respiration and ATP biosynthesis (Turner et al., 2008; Yin et al., 2008; Kim et al., 2009; Kumar et al., 2015; Yao et al., 2015).

BBR suppresses lipogenesis and promotes lipolysis in liver cells and adipocytes through AMPK activation (Kim et al., 2009; Wang et al., 2016). However, whether or not AMPK is essential for the glucose-lowering activity of BBR remains controversial. For example, a report showed that in HepG2 cells and C2C12 muscle cells, BBR stimulated glucose consumption in an AMPK-independent manner, as compound C, AMPKα small interfering RNA (siRNA), or dominant negative (DN)-AMPKα did not block the activity of BBR (Xu et al., 2014). A recent report also proved that AMPK was not required for BBR to promote glucose uptake in fibroblasts (Cok et al., 2011). However, several other studies showed that by using compound C (Cheng et al., 2006) or iodotubercidine (Kim et al., 2007), the glucose consumption- or uptake-stimulating activities of BBR in muscle cells or adipocytes were blocked, either partially or completely.

In this study, in order to clarify the role of AMPK in the glucose-lowering activity of BBR, we knocked-out (KO) AMPKα1 in HepG2 cells (*AMPKα1^-/-^*) and found that the activities of BBR on cellular glucose and lipid metabolism were completely abolished. Our results clearly prove that AMPKα1 is essential for BBR to promote glucose and lipid metabolism in HepG2 cells.

## Materials and Methods

### Reagents and Kits

Dimethyl sulfoxide (DMSO, D2438), berberine (BBR, purity over 98%, B3251), oleic acid (OA, purity over 99%, O1383), bovine serum albumin (BSA, B2064), sodium L-lactate (L7022), and sodium pyruvate (P8574) were purchased from the Sigma-Aldrich Co. LLC (St. Louis, MO). Fetal bovine serum (FBS, #10100154), minimum essential medium (MEM, #11095080), protease inhibitors (#78442), and kits for cell protein extraction (#89900) and quantification (#23225) were purchased from Thermo Fisher Scientific (China) Inc (Shanghai, China). The rabbit or mouse monoclonal antibodies against AMPKα (#2793), phosphorylated-AMPKα (p-AMPKα) (Thr172) (#2535), AMPKα1 (#2795), p-AMPKα1 (Ser485) (#2537), AMPKα2 (#2757), p-ACC (#3661), P-Raptor (#2083), and β-actin (ACTB) (#4970) were purchased from Cell Signaling Technology, Inc (Danvers, MA). The antibody against CD36 (ab133625) was purchased from Abcam (Cambridge, MA). The AMPKα1 CRISPR/Cas9 KO Plasmid (sc-400104), AMPK 1 HDR (homology-directed repair) Plasmid (sc-400104-HDR), and puromycin dihydrochloride (sc-108071) were purchased from Santa Cruz Biotechnology, Inc (Dallas, TX). The Lipofectamine^™^ 3000 Transfection Kit (L3000150) and the Amplex^™^ Red Glucose/Glucose Oxidase Assay Kit (A22189) were purchased from Invitrogen (Grand Island, NY). The Cyclex^®^ AMPK Kinase Assay Kit (cy-1182) was purchased from Cyclex Co. Ltd. (Nagano, Japan). The commercially available kits for the assay of L-lactate (GX8127T) and glucose (GS121T) were purchased from Beijing Strong Biotechnologies, Inc (Beijing, China). The Steatosis Colorimetric Assay Kit (10012643) was purchased from Cayman Chemical (Ann Arbor, MI) and the Tissue/Cell Triglyceride (TG) Assay Kit (E1013) was from Applygen Technologies Inc. (Beijing, China). Reagents and kits for cellular RNA extraction (#9109), reverse transcription (RT) (RR037A), and quantitative real-time PCR (RR820A) were purchased from Takara Bio Inc (Shiga, Japan).

### Cell Culture, Transfection, and Treatment

HepG2 cells were routinely cultured in MEM + 10% FBS at 37°C in a 5% CO_2_ incubator. Before transfection, cells were trypsinized with 0.25% trypsin-ethylenediaminetetraacetic acid (EDTA) and seeded onto six-well plates with 1×10^6^/well. When the cells reached about 70–80% confluence, the transfection experiment was performed. Briefly, for one well of the six-well plate, 7.5 μl of the Lipofectamine^™^ 3000 reagent was added to 125 μl of serum-free MEM and mixed well. Meanwhile, 1 μg of the AMPKα1 CRISPR/Cas9 KO Plasmid and 1 μg of the AMPKα1 HDR Plasmid were diluted with 125 μl of serum-free MEM, then, 4 μl of the P3000^™^ reagent was added and mixed well. The diluted plasmids were then added to the diluted transfection reagent at 1:1 ratio and mixed well. After incubation for 5 min at room temperature, the master mix (250 μl) was added to cells. CRISPR/Cas9 plasmid induced double-strand breaks (DSB) of target gene (AMPKα1). Target-specific HDR Plasmid provided a DNA repair template for a DSB and enabled the insertion of a Red Fluorescent Protein (RFP) gene to visually confirm transfection and an antibiotic resistance gene (puromycin) for selection of cells containing a successful CRISPR/Cas9 double-strand break.

After 48 h of transfection, cells were trypsinized, diluted, and cultured in 96-well plate, and stable monoclonal transfectants were selected in medium containing 0.5 μg/ml of puromycin for about 1 month. DNA transfection was confirmed by visualization of red fluorescence under an Olympus IX71 inverted fluorescence microscope (Olympus Corporation, Tokyo, Japan). And the disruption of target gene was confirmed by DNA sequencing (Invitrogen) and western blot.

The HepG2 *AMPKα1^-/-^* cells were maintained in MEM + 10% FBS containing 2 μg/ml puromycin. Together with wild type, the cells were seeded onto 6-well plates, 24-well plates (4×10^5^/well), or 96-well plates (5×10^4^/well) before experiments and starved in serum-free MEM for 24 h before treatment. BBR (dissolved as 10 mM in DMSO) was used to treat the cells at indicated concentrations for 24 h in serum-free MEM. In some cases, OA (0.6 mM) dissolved in phosphate buffered saline (PBS) plus 0.5% BSA was also used to treat the cells, either alone or in combination with BBR.

### Western Blot

After treatment, cells cultured in six-well plates were rinsed with PBS and total proteins were extracted as described before (Zhang et al., 2018). For each sample, about 15 μg of proteins were subjected to 10% SDS-PAGE, and protein bands were transferred onto PVDF membranes (Millipore, Billerica, MA) through a Wet Transfer Cell (Bio-Rad, Hercules, CA). For the detection of target protein expression levels, membranes were probed with specific rabbit or mouse monoclonal antibodies with ACTB as an internal control. After washing and incubation with appropriate secondary antibodies, the bands were visualized with a Chemi-Luminescent Horseradish Peroxidase (HRP) Kit (Millipore). The signal intensities were quantified with Gel-Pro Analyzer 4.0 software (Media Cybernetics, Inc, Rockville, MD).

### AMPK Activity Assay

After treatment, cells were lysed and AMPK activities were measured according to the protocol of the kit. Briefly, samples were added to a plate which was coated with an AMPK-substrate, and the reactions were started by adding of Mg^2+^ and ATP. After proper incubation and washing, a monoclonal antibody specific for the phosphorylated form of the substrate was added. After another round of incubation and washing, a HRP-conjugated secondary antibody was added to the well. The color was developed by a chromogenic substrate and the signals were measured densitometrically at 450 nm. The AMPK inhibitor compound C was used as an “inhibitor control.” After subtracting OD450 of compound C-treated parallel samples, the OD450 values of the samples were used as the “relative AMPK activity” and were presented as percentage of control cells.

### Glucose Consumption and Lactate Release Assay

The cells were seeded onto 96-well plates and there were five to six replicate wells for each treatment. After treatment, culture mediums were collected and centrifuged at 1,000 rpm for 5 min. Glucose levels were assayed by a kit which was based on the glucose oxidase method, and glucose consumption was calculated as described in our previous report (Shan et al., 2013). Meanwhile, the concentration of L-lactate in the supernatant was also determined by a commercial kit according to the protocol.

### Gluconeogenesis Assay

The cells were seeded onto 24-well plates and there were four replicate wells for each treatment. After treatment, cells were washed with PBS and glucose production medium which contained 20 mM of sodium L-lactate and 2 mM of sodium pyruvate was added (100 μl/well). After 3.5 h of incubation, glucose levels were determined by a kit, normalized to protein concentrations, and presented as fold of vehicle-treated wild type cells, which was designated as 1 (Zhang et al., 2012).

### Oil Red O (ORO) Staining and Intracellular TG Assay

The cells were seeded onto 24-well plates with three replicate wells for each treatment; after treatment, cells were subjected to ORO staining as described before (Wang et al., 2016). After photograph, the wells were let dry, ORO was extracted by the Lipid Droplets Assay Dye Extraction Solution and the absorbance values at 490 nm (OD490) were read by a plate reader (PerkinElmer, Inc, Waltham, MA). In parallel experiments, intracellular TG levels were determined by a kit and normalized to protein concentrations.

### Real-Time RT-PCR

The cells were seeded onto six-well plates, after treatment, total cellular RNAs were isolated and 1 μg was used as a template to reverse transcribed into cDNAs in a 20 μl of reaction system as described previously (Shan et al., 2013). For quantitative PCR, a 50 μl of reaction system was set up which contained 25 μl of SYBR Premix Ex Taq II (2×), 2 μl of forward primer (**Table 1**), 2 μl of reverse primer (**Table 1**), 1 μl of ROX Reference Dye (50×), 4 μl of cDNA, and 16 μl of water. The reactions were performed on an Applied Biosystems 7500 Fast Real-Time PCR System (Foster City, CA) and the conditions were as following: 95°C, 30 sec, followed by 40 cycles of 95°C, 5 sec + 60°C, 30 sec. Relative quantification of the levels of target genes (**Table 1**) was conducted by using ACTB as an internal control.

**Table 1:** Primers for real-time PCR (5′ to 3′).

Genes	Sense primer	Anti-sense primer
CHREBP	agagacaagatccgcctgaa	cttccagtagttccctcca
FAS	gacatcgtccattcgtttgtg	cggatcaccttcttgagctcc
ACTB	tcaacaccccagccatgta	agtacggccagaggtgtacg

### Statistical Analysis

After validation of the test for homogeneity of variance (Bartlett’s test), results were examined by one-way ANOVA followed by the Newman-Keuls test for multiple comparisons. *P* < 0.05 was considered to be statistically significant.

## Results

### BBR Stimulates Glucose Consumption and Inhibits Gluconeogenesis Through AMPKα1 Activation in HepG2 Cells

**Figure 1:**
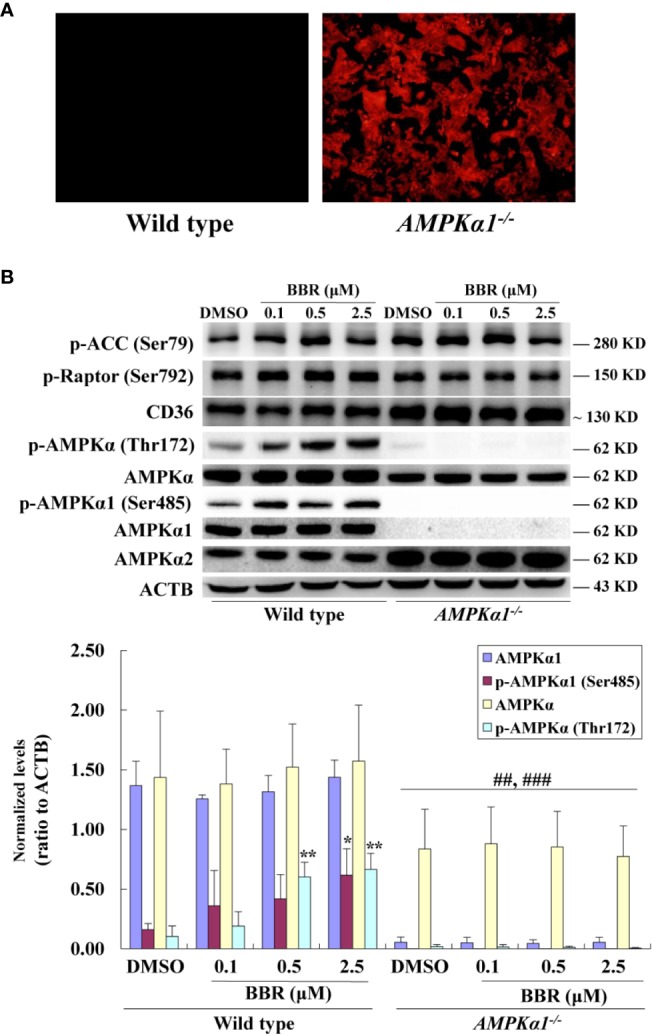
Effects of BBR on AMPK phosphorylation in wild type and *AMPKα1^-/-^* HepG2 cells. The *AMPKα1* gene of HepG2 cells was knocked-out by co-transfection of AMPKα1 CRISPR/Cas9 KO Plasmid and AMPK 1 HDR Plasmid as described in the materials and methods. Monoclonal transfectants were selected by puromycin and DNA transfection was visualized by red fluorescence **(A)** (×100). After serum starvation, cells were treated with DMSO or BBR for 24 h as indicated; cell total proteins were extracted for western blot analysis of the expression levels of target proteins, which were normalized to that of ACTB and plotted as indicated **(B)**. Representative images of three separate experiments are presented. Values in **(B)** are mean ± SD of three separate experiments; *p < 0.05, **p < 0.01 *vs.* that of wild type cells treated with DMSO; ^##^*p* < 0.01, ^###^*p* < 0.001 *vs.* that of same treated wild type cells.

In order to clarify whether or not AMPK is indispensable for BBR to stimulate glucose metabolism, *AMPKα1^-/-^* HepG2 cells were generated by disruption of the *AMPKα1* gene and stable monoclonal transfectants were selected. As shown in **Figure 1A**, while wild type HepG2 cells had no fluorescence, the *AMPKα1^-/-^* HepG2 cells presented a strong red fluorescence due to co-transfection of an HDR plasmid, which verified the success of DNA transfection.

The disruption of *AMPKα1* gene was verified by DNA sequencing (data not shown) and western blot analysis of target proteins. As shown in **Figure 1B**, the protein of AMPKα1 completely disappeared in the *AMPKα1^-/-^* HepG2 cells. In wild type HepG2 cells, BBR had no influence on the level of AMPKα1, but stimulated its phosphorylation at Ser485 significantly (*p* < 0.05 *vs.* DMSO). In *AMPKα1^-/-^* HepG2 cells, the phosphorylation of AMPKα1 protein was not observed and the activity of BBR was completely abolished (*p* < 0.001 *vs.* wild type).

When total AMPKα was detected, the result showed that its protein level declined to about 45–50% of wild type (*p* < 0.01) in *AMPKα1^-/-^* HepG2 cells (**Figure 1B**). BBR had no influence on the protein level of total AMPKα in wild type or *AMPKα1^-/-^* HepG2 cells, but increased its phosphorylation at Thr172 significantly in wild type cells (*p* < 0.05 or *p* < 0.01 *vs.* DMSO). In *AMPKα1^-/-^* HepG2 cells, the phosphorylation of total AMPKα declined to less than 10% of wild type cells (*p* < 0.001) and the stimulating activity of BBR on AMPKα phosphorylation was also abolished (*p* < 0.001 *vs.* wild type) (**Figure 1B**). Consistent with this, BBR increased the expression of P-ACC and P-Raptor compared to the vehicle group in wild-type but not *AMPKα1^-/-^* HepG2 cells (**Figure 1B**).

**Figure 2:**
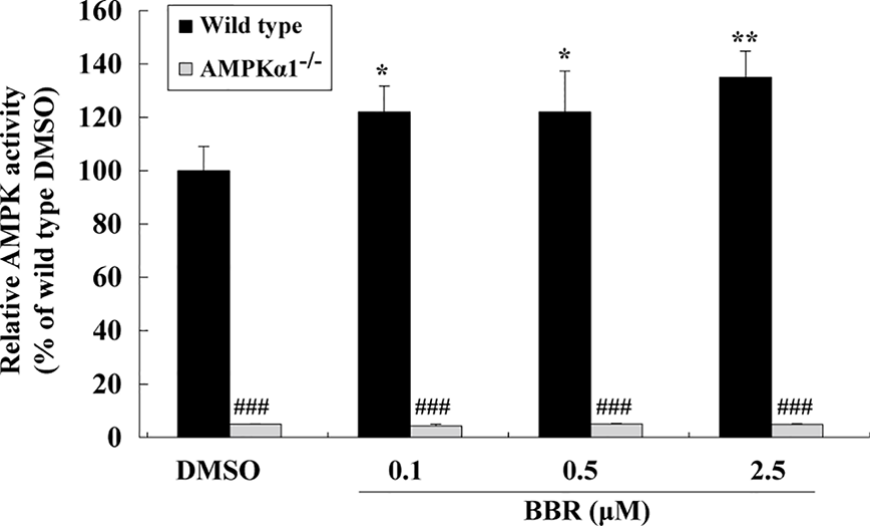
Effects of BBR on AMPK activity in wild type and *AMPKα1^-/-^* HepG2 cells. Cells were treated as described in **Figure 1B**. After treatment, cells were harvested; relative AMPK activities were measured by a kinase assay kit and presented as percentages of wild type cells treated with DMSO. Values are mean ± SD of three separate experiments; *p < 0.05, **p < 0.01 *vs.* that of wild type cells treated with DMSO; ^###^*p* < 0.001 *vs.* that of same treated wild type cells.

The AMPK activity assay was used to verify the effects of BBR and results were in agreement with those of western blot. As shown in **Figure 2**, BBR greatly induced AMPK activity in wild type HepG2 cells (*p* < 0.05 or *p* < 0.01 *vs.* DMSO), and the activity was completely blocked by *AMPKα1* gene KO (*p* < 0.001 *vs.* wild type). Taken together, the above results proved that *AMPKα1* gene was successfully knocked-out in HepG2 cells and the stimulating activity of BBR was blocked.

**Figure 3:**
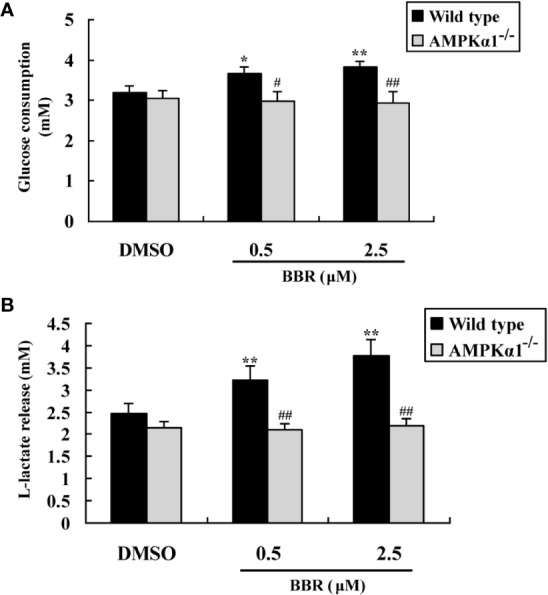
Effects of BBR on cellular glucose consumption and lactate release. After serum starvation, wild type and *AMPKα1^-/-^* HepG2 cells were treated with DMSO or BBR for 24 h; glucose levels in the culture supernatants were assayed for the determination of glucose consumption **(A)**, and the concentrations of L-lactate were also determined **(B)**. Values are mean ± SD of three separate experiments; *p < 0.05, **p < 0.01 *vs.* that of wild type cells treated with DMSO; ^#^*p* < 0.05, ^##^*p* < 0.01 *vs.* that of wild type cells treated with BBR at a same concentration.

**Figure 3A** showed that BBR greatly enhanced glucose consumption in wild type HepG2 cells after 24 h of treatment (*p* < 0.05 or *p* < 0.01 *vs.* DMSO). However, in *AMPKα1^-/-^* HepG2 cells, the stimulating effect of BBR on cellular glucose consumption was completely abolished (*p* < 0.05 or *p* < 0.01 *vs.* wild type). As BBR was reported to stimulate glucose consumption through induction of glycolysis (Yin et al., 2008), we determined the concentrations of L-lactate in the culture supernatant after treatment. As shown in **Figure 3B**, the stimulating effect of BBR on lactate release in wild type HepG2 cells was completely blocked when *AMPKα1* gene was knocked-out (*p* < 0.01 *vs.* wild type).

**Figure 4:**
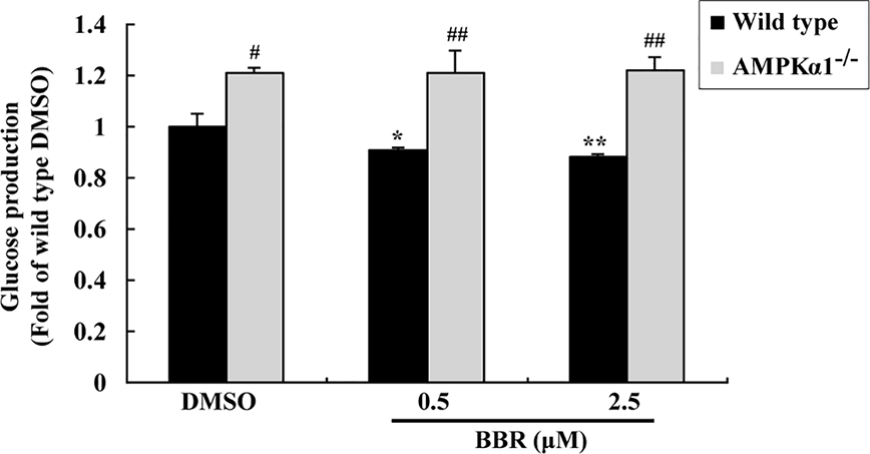
Effects of BBR on cellular gluconeogenesis. After serum starvation, wild type and *AMPKα1^-/-^* HepG2 cells were treated with DMSO or BBR for 24 h; gluconeogenesis was determined by using a glucose production medium and normalized to protein contents as described in *Materials and Methods*. Values are mean ± SD of three separate experiments; *p < 0.05, **p < 0.01 *vs.* that of wild type cells treated with DMSO; ^#^*p* < 0.05, ^##^*p* < 0.01 *vs.* that of same treated wild type cells.

The activation of AMPK was able to suppress gluconeogenesis in hepatocytes (Yadav et al., 2017), therefore, we determined glucose production after BBR treatment. As shown in **Figure 4**, in wild type HepG2 cells, BBR reduced glucose production significantly after 24 h of treatment (*p* < 0.05 or *p* < 0.01 *vs.* DMSO). The baseline glucose production of the *AMPKα1^-/-^* HepG2 cells was greatly elevated as compared to wild type cells (*p* < 0.05). Furthermore, the suppressing effect of BBR on gluconeogenesis was completely abolished after disruption of the *AMPKα1* gene (*p* < 0.01 *vs.* wild type).

In summary, the above results prove that in HepG2 cells, the activation of AMPKα1 is essential for BBR to stimulate glucose consumption and inhibit gluconeogenesis.

### BBR Promotes Lipid Metabolism Through AMPKα1 Activation in HepG2 Cells

**Figure 5:**
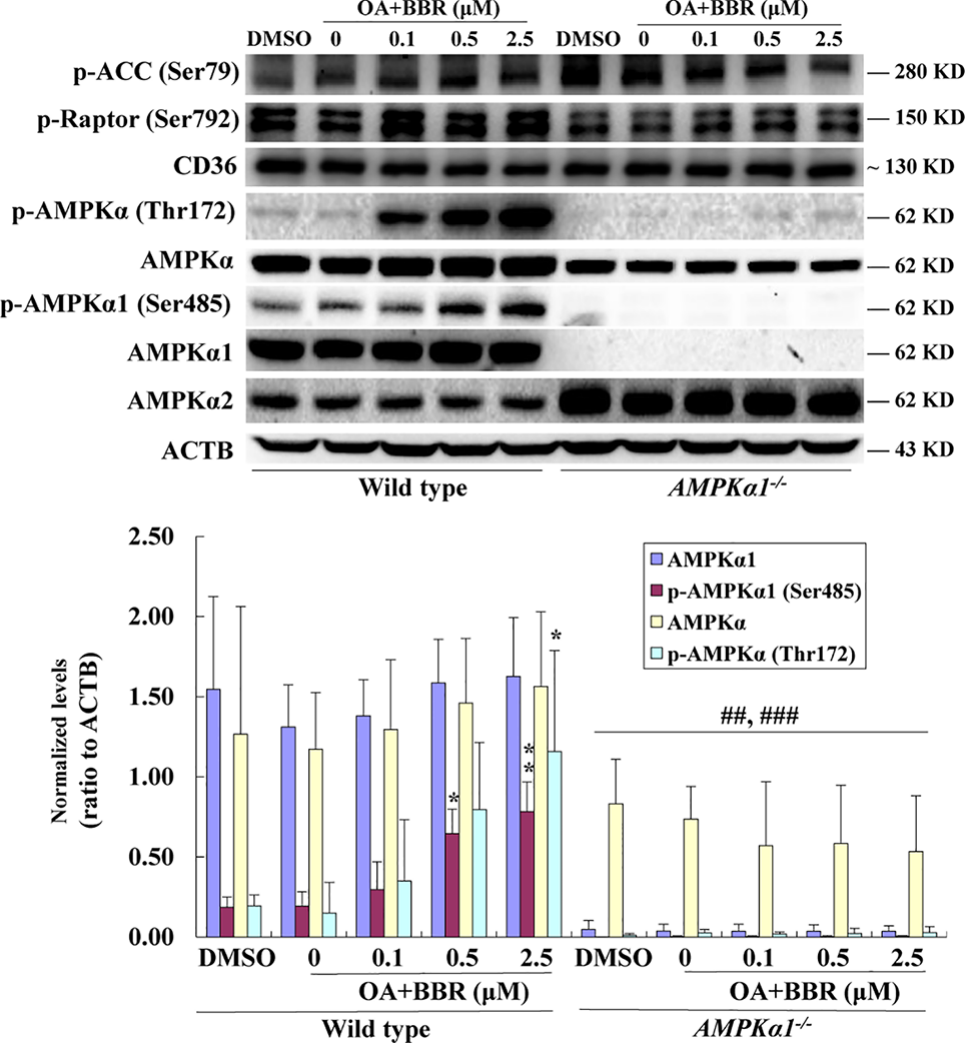
Effects of BBR on AMPK phosphorylation in wild type and *AMPKα1^-/-^* HepG2 cells treated with OA. After serum starvation, cells were treated with DMSO, OA (0.6 mM), or OA + BBR for 24 h as indicated. Cell total proteins were extracted for western blot analysis of the expression levels of target proteins, which were normalized to that of ACTB and plotted as indicated. Representative images of three separate experiments are presented. Values are mean ± SD of three separate experiments; *p < 0.05, **p < 0.01 *vs.* that of wild type cells treated with OA alone; ^##^*p* < 0.01, ^###^*p* < 0.001 *vs.* that of same treated wild type cells.

**Figure 6:**
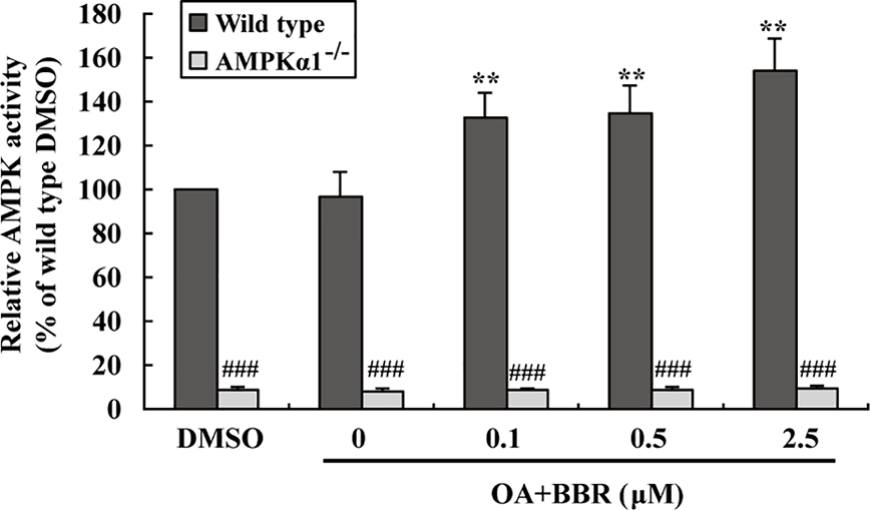
Effects of BBR on AMPK activity in wild type and *AMPKα1^-/-^* HepG2 cells treated with OA. Cells were treated as described in **Figure 5**. After treatment, cells were harvested; relative AMPK activities were measured and presented as percentages of wild type cells treated with DMSO. Values are mean ± SD of three separate experiments, **p < 0.01 *vs.* that of wild type cells treated with OA alone, ^###^*p* < 0.001 *vs.* that of same treated wild type cells.

In order to investigate the effects of BBR on AMPK in the presence of OA, wild type and *AMPKα1^-/-^* HepG2 cells were co-administered with OA and BBR for 24 h, and cell proteins were extracted for western blot analysis. As shown in **Figure 5**, OA or BBR treatment did not influence the protein level of AMPKα1 or total AMPKα in wild type or *AMPKα1^-/-^* HepG2 cells. In wild type cells, BBR greatly stimulated the phosphorylation of AMPKα1 and total AMPKα (**Figure 5**) and enhanced AMPK activity (**Figure 6**) in the presence of OA (*p* < 0.01 *vs.* DMSO). In *AMPKα1^-/-^* HepG2 cells, the AMPKα1 protein and its phosphorylation disappeared (*p* < 0.001 *vs.* wild type) and the protein level of total AMPKα declined to about 50% of wild type cells (*p* < 0.01) (**Figure 5**). The phosphorylation of total AMPKα (**Figures 1B**, **5**) and AMPK activity (**Figures 2**, **6**) of *AMPKα1^-/-^* HepG2 cells were greatly reduced to less than 10% of wild type cells (*p* < 0.001) with or without OA. In *AMPKα1^-/-^* HepG2 cells treated with OA, the stimulating effects of BBR on AMPKα1 phosphorylation (**Figure 5**), total AMPKα phosphorylation (**Figure 5**), and AMPK activity (**Figure 6**) were totally abolished (*p* < 0.001 *vs.* wild type). Consistent with this, BBR increased the expression of P-ACC and P-Raptor compared to the OA-treated group in wild-type but not *AMPKα1^-/-^* HepG2 cells (**Figure 5**).

**Figure 7:**
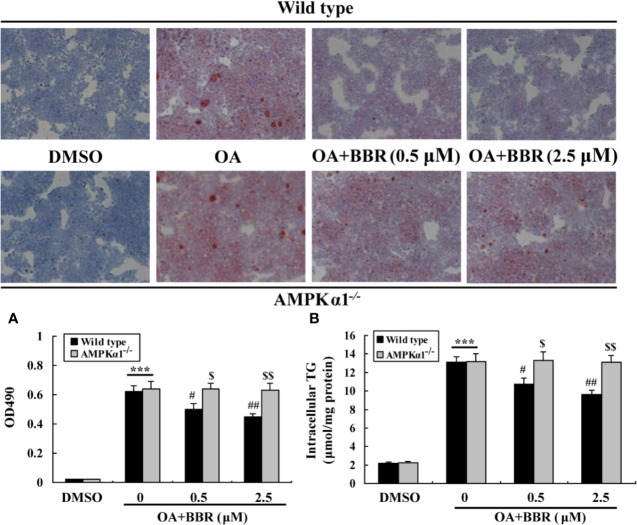
Effects of BBR on intracellular lipid accumulation. Cells were treated as indicated for 24 h, and cellular steatosis was determined by ORO staining (×100) (upper panel) followed by quantification (lower panel) **(A)**. In parallel experiments, intracellular TG were determined and normalized to protein contents **(B)**. Values are mean ± SD of three separate experiments; ***p < 0.001 *vs.* that of DMSO; ^#^*p* < 0.05, ^##^*p* < 0.01 *vs.* that of wild type cells treated with OA alone; ^$^*p* < 0.05, ^$$^*p* < 0.01 *vs.* that of wild type cells treated with OA+BBR at a same concentration.

**Figure 8:**
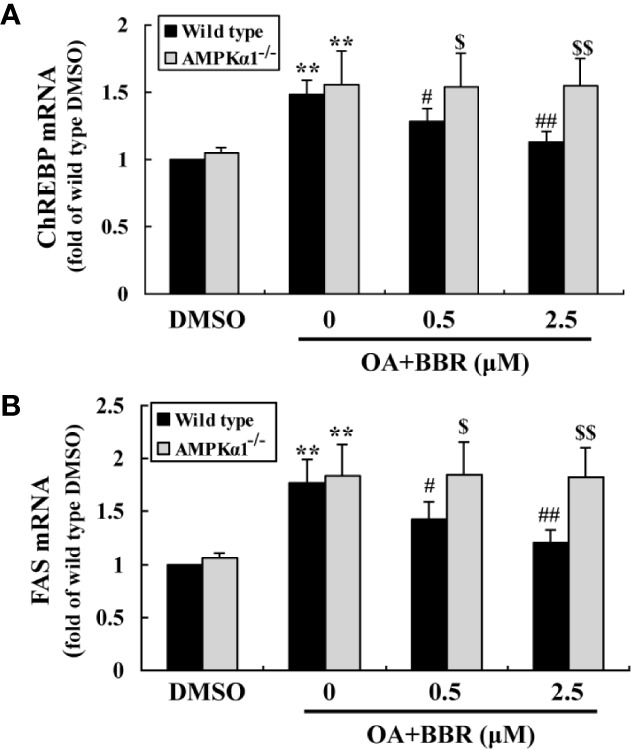
Effects of BBR on the mRNA expression levels of lipogenic genes. Cells were treated as indicated for 24 h. After treatment, cell total RNAs were extracted for real-time RT-PCR analysis of the mRNA expression levels of ChREBP **(A)** and FAS **(B)**, which were normalized to ACTB and presented as fold of wild type cells treated with DMSO. Values are mean ± SD of three separate experiments; **p < 0.01 *vs.* that of DMSO; ^#^*p* < 0.05, ^##^*p* < 0.01 *vs.* that of wild type cells treated with OA alone; ^$^*p* < 0.05, ^$$^*p* < 0.01 *vs.* that of wild type cells treated with OA+BBR at a same concentration.

OA treatment induced lipid accumulation in wild type and *AMPKα1^-/-^* HepG2 cells, as determined by ORO staining (**Figure 7A**) and intracellular TG assay (**Figure 7B**) (*p* < 0.001 *vs.* DMSO). In wild type HepG2 cells, BBR administration greatly reduced lipid accumulation, as indicated by an improvement of ORO staining and a reduction of intracellular TG (*p* < 0.05 or *p* < 0.01 *vs.* OA alone). The anti-lipid effect of BBR was totally blocked in *AMPKα1^-/-^* HepG2 cells (*p* < 0.05 or *p* < 0.01 *vs.* wild type). Accompanied with lipid accumulation, cellular mRNA expression levels of lipogenic genes like carbohydrate responsive element-binding protein (ChREBP) and fatty acid synthase (FAS) increased greatly (*p* < 0.01 *vs.* DMSO) (**Figure 8**). Co-administration of BBR prevented the up-regulation of ChREBP and FAS induced by OA (*p* < 0.05 or *p* < 0.01 *vs.* OA alone), and the inhibitory effects were abolished upon AMPKα1 knockout (*p* < 0.05 or *p* < 0.01 *vs.* wild type). Taken together, the above results prove that in HepG2 cells, AMPKα1 is also essential for BBR to improve lipid metabolism.

## Discussion

In the current study, we report for the first time that natural product BBR promotes glucose and lipid metabolism in HepG2 cells through activating AMPKα1. Our study distinguishes from previous reports, as gene KO technology is applied and AMPKα1 is selectively deleted, which make our results solid.

In AMPKα1 deficient HepG2 cells, the protein expression of AMPKα1 disappeared and the stimulating effect of BBR on its phosphorylation was completely abolished. In addition, we noticed that the phosphorylation of total AMPKα was potently inhibited and the stimulating effect of BBR on it were totally abolished in AMPKα1 deficient HepG2 cells, and the results were verified by kinase activity assay. The α subunit of AMPK has two isoforms, which are α1 and α2 (Steinberg and Kemp, 2009). The remaining AMPKα in AMPKα1 deficient HepG2 cells ought to be AMPKα2, and our results implied that deletion of AMPKα1 might have negative effect on AMPKα2 activation. As far as we know, there are no reports showing the significance of single subtype of AMPK alpha to BBR by conducting CRISPR/Cas9 KO *in vitro*. Studies on BBR’s action using siRNA knockdown for AMPKalpha1 subtype are also very few. Similar findings were reported in a previous study (Jang et al., 2017), in which AMPKα1 siRNA was used to transfect 3T3-L1 preadipocytes, and the results showed that at the highest siRNA dose, the protein level of total AMPKα declined significantly, while that of p-AMPKα totally disappeared. These results were in agreement with our observation.

The inhibitory effects of BBR on OA-induced up-regulation of lipogenic genes and intracellular lipid accumulation were blocked in AMPKα1 deficient HepG2 cells. These results are in agreement with previous reports in which compound C (Zhou et al., 2018) or RNA silencing (Jang et al., 2017) is used and support the inhibitory role of AMPK on lipogenesis. Upon activation, AMPK is associated with a down-regulation of lipogenic genes and an up-regulation of lipolytic genes, which will reduce fat synthesis while promote lipolysis (Lee et al., 2006; Dihingia et al., 2018).

The most important finding of this study is that the stimulating activity of BBR on cellular glucose consumption is dependent on AMPKα1 activation in HepG2 cells. To our knowledge, this is the first report which clearly demonstrates that AMPK plays a critical role in the glucose-lowering effect of BBR by using a gene KO model. As an end-product of glycolysis, the level of L-lactate was increased in the culture supernatant after BBR treatment and this effect was abolished when AMPKα1 was knockout. Glycolysis is modulated by a set of enzymes and factors, which include AMPK (Li et al., 2015). AMPK was reported to stimulate glycolysis through activating phosphofructokinase (PFK), a critical enzyme of cellular glycolysis (Marsin et al., 2000; Yan et al., 2017). It is possible that BBR may influence the critical enzymes of glycolysis through activating AMPK, which merits further investigation. In addition to glycolysis, AMPK is able to stimulate cellular glucose uptake through induction of membrane translocation of glucose transporters (GLUTs) (Lee et al., 2012). This activity of AMPK may also contribute to the stimulating effect of BBR on glucose utilization (Cheng et al., 2006; Kim et al., 2007; Cok et al., 2011; Kumar et al., 2015; Yao et al., 2015). Taken together, results of our study and other researchers demonstrate that AMPK is essential for the glucose-lowering activity of BBR.

In addition to stimulation of glucose consumption, BBR also inhibits gluconeogenesis of HepG2 cells, and this inhibitory effect is blocked in AMPKα1 deficient cells. Our results suggest that BBR suppresses liver glucose production in an AMPK-dependent manner, which is in agreement with previous studies (Zhang et al., 2012). After BBR treatment and AMPK activation, key genes involved in hepatic gluconeogenesis such as forkhead transcription factor O1 (FOXO1), phosphoenolpyruvate carboxykinase (PEPCK), and glucose-6-phosphatase (G6Pase) are down-regulated (Xia et al., 2011; Wang et al., 2016), and this will result in the reduction of glucose production and improvement of glucose homeostasis.

BBR increased the expression of P-ACC and P-Raptor in the wild type but not *AMPKα1^-/-^* HepG2 cells, in the presence or absence of OA (**Figures 1B**, **5**). It seems that BBR may inhibit the activity of ACC through AMPKα1 as well.

BBR didn’t influence the CD36 expression in HepG2 cells of both wild-type and *AMPKα1^-/-^* background, compared to the vehicle and OA-treated group respectively (**Figures 1B**, **5**). The effects of BBR on CD36 seem controversial in liver. Choi et al. reported that BBR up-regulated hepatic CD36, increased fatty acid uptake by liver cells and caused hepatocellular lipid accumulation and fatty liver (Choi et al., 2017). However, their results are conflict with clinical observations, in which fat content in liver is largely reduced (Yan et al., 2015). For CD36, there is also a report indicating that BBR down-regulates CD36 in mouse liver (Sun et al., 2017). Moreover, high dose of 10–25 μM was used in the mechanism study of BBR in this research (Choi et al., 2017), which was not reachable *in vivo* and would be discussed next.

Although the current study is performed in cultured cells, we believe that similar results may be obtained *in vivo*. After oral administration, BBR is poorly absorbed and the blood concentration is low (Shan et al., 2013). However, recent studies prove that BBR and its main phase-I metabolites are able to be enriched in organs like liver, kidney, and lung, and the tissue concentration of BBR is greatly higher than its blood concentration (Tan et al., 2013; Yan et al., 2015). For example, after single dose oral administration in rats, the liver concentration of BBR is more than 50-fold of the blood concentration (Yan et al., 2015). The concentrations of BBR used in our experiments are 0.1–2.5 μM (Zhang et al., 2014). We find that as low as 0.1–0.5 μM of BBR is able to activate AMPK and improve glucose and lipid metabolism in HepG2 cells, and these concentrations are reachable *in vivo* in liver after oral administration (Tan et al., 2013; Yan et al., 2015).

In conclusion, our study demonstrates that AMPKα1 is essential for BBR to improve glucose and lipid metabolism in HepG2 cells. Our results may help to further understand the molecular mechanisms and signaling network of BBR and may provide further scientific evidence for the clinical application of this promising natural product to treat metabolic disorders.

## Data Availability Statement

The raw data supporting the conclusions of this manuscript will be made available by the authors, without undue reservation, to any qualified researcher.

## Author Contributions

GR designed and performed the experiments, analyzed the data, and wrote the manuscript. J-HG performed the experiments and analyzed the data. Y-ZQ transfected and obtained AMPKα1 knockout cells. J-DJ and W-JK oversaw the project, designed the experiments, analyzed the data, and wrote the manuscript.

## Funding

This work was supported by the CAMS Innovation Fund for Medical Sciences (CIFMS) (2016-I2M-1-011) and the National Natural Science Foundation of China (81302823 and 81621064).

## Conflict of Interest

The authors declare that the research was conducted in the absence of any commercial or financial relationships that could be construed as a potential conflict of interest.
